# Oridonin induces Mdm2‐p60 to promote p53‐mediated apoptosis and cell cycle arrest in neuroblastoma

**DOI:** 10.1002/cam4.2393

**Published:** 2019-07-24

**Authors:** Han‐Qing Zhu, Chao Zhang, Zhu‐Ying Guo, Jun‐Mei Yang, Jia‐Hui Guo, Chen Chen, Qiang‐Hua Yao, Feng Liu, Quan‐Wu Zhang, Feng‐Hou Gao

**Affiliations:** ^1^ Department of Oncology, Shanghai 9th People's Hospital Shanghai Jiao Tong University School of Medicine Shanghai China; ^2^ Department of Geriatrics, Shanghai 9th People's Hospital Shanghai Jiao Tong University School of Medicine Shanghai China; ^3^ Department of Clinical Laboratory Children's Hospital Affiliated to Zhengzhou University Zhengzhou China; ^4^ Department of Pediatrics The First Affiliated Hospital of Zhengzhou University Zhengzhou China; ^5^ Department of Pathology Zhengzhou Central Hospital Affiliated to Zhengzhou University Zhengzhou China

**Keywords:** apoptosis, cell cycle arrest, Mdm2‐p60, Oridonin, p53, ROS

## Abstract

Oridonin could induce NB (neuroblastoma) cells growth inhibition by inducing apoptosis and cell cycle arrest, and the molecular mechanisms behind the effects deserve to be further explored. Here, oridonin was confirmed to cause the reactivation of p53 (cellular tumor antigen p53) to promote the expression of a series of apoptosis‐ and cell cycle arrest‐related proteins for the biological effects. During the process, oridonin relied on the caspase activation to cleave p53‐induced Mdm2 (E3 ubiquitin‐protein ligase Mdm2) to generate Mdm2‐p60. The generation of Mdm2‐p60 stabilized p53, and resulted in p53 accumulation for p53 continuous activation. In our research, it was also found that the reactivation of p53 induced by oridonin was closely related with the generation of ROS (reactive oxygen species). Taken together, these findings explain that oridonin exerts its anticancer activity partially by targeting the Mdm2‐p53 axis in NB cells, which lay an experimental base for future research of exploring the effects and molecular mechanisms of oridonin.

## 1 INTRODUCTION

NB (neuroblastoma), which origins from the neural crest, is the malignant solid tumor of great heterogeneity in children and babies, whose age of onset is commonly from 1 to 5 years old.^1^ Although great progress has been made in the surgery, chemotherapy, and radiation therapy for NB over the past 20 years, the treatment of the elderly and end‐stage patient whose 5‐year survival rate is less than 30% is still a tough problem to be solved.^1^ Chemotherapy is preferred because of the difficulty of executing surgery and radiation therapy for the advanced patients. To provide new hope for the chemotherapy of NB, it is necessary to find effective target molecules in NB and seek candidate compounds or their derivatives which target these molecules.

Cell apoptosis inhibition and cell cycle checkpoint runaway are the hallmarks of tumor development and progression, and are significant features of cancer.^2^ The tumor suppressor p53 usually functions as a transcription factor. The activation of p53 induced by external stimulation can suppress the proliferation of cancer cells, because it can promote the expression of a series of related proteins to induce cell apoptosis and cell cycle arrest.^3^, ^4^ However, the inactivation or low expression of p53 and mutations of *TP53 (tumor protein p53)* frequently occur in human cancers of different types.^5^, ^6^ In NB, *TP53* rarely mutates and the signaling pathways on the downstream of p53 are intact.^7^ Meanwhile, p53 inactivation is considered to be the most frequent mechanism of the drug resistance in NB cells.^8^ Furthermore, it has already been confirmed that reactivation of p53 in NB cells can induce cell apoptosis through the signaling pathways on the downstream of p53.^9^, ^10^ Based on these findings, exploring small molecular compounds which can reactivate p53 to induce NB cells apoptosis and cell cycle arrest may provide a promising solution for the treatment of NB.^9^, ^11^, ^12^

Oridonin is a kind of active diterpenoid derived from traditional Chinese medicine.^13^ It has a wide range of biological effects, such as anticancer, antibacterial, and anti‐inflammatory activities.^14^ And, many oridonin derivatives have been designed and synthesized.^14^, ^15^ Oridonin has strong anticancer activity that can extend the survival period of models of transplanted human esophageal and gastric tumor in mice.^16^ As reported in the literature, oridonin can induce apoptosis or cell cycle arrest in pancreatic cancer, gastric cancer, liver cancer, prostate cancer, and colorectal cancer cells.^17^, ^18^, ^19^, ^20^ It is especially crucial that several studies have shown that during the apoptosis of cancer cells induced by oridonin or its derivatives, p53 is reactivated and the proteins on the downstream of p53 are also altered.^20^, ^21^ For example, oridonin induces the growth inhibition and apoptosis of gastric cancer cells by regulating the expression and function of p53^22^; the anticancer effects of oridonin on colon cancer cells are mediated through BMP7/p38 MAPK/p53 signaling pathway^23^; Geridonin, a derivative of oridonin, in combination with paclitaxel can lead to the accumulation of p53, and further apoptosis of gastric cancer cells by the mitochondrial pathway.^24^ Furthermore, the apoptosis and autophagy of murine fibrosarcoma cells induced by oridonin are also p53‐dependent.^25^ These preliminary studies show that oridonin may exhibit anticancer activity by reactivating p53, but the molecular mechanisms by which oridonin regulates p53 have not been elucidated in detail.

Our previous studies have shown that oridonin enhances the anticancer activity of NVP‐BEZ235 against NB cells through autophagy.^13^ And, it has also been proved that oridonin can also generate ROS to sensitize NB cells to TRAIL‐induced apoptosis.^26^ At present, we investigate the effects of oridonin on NB cells and further explore the detailed molecular mechanisms. We find that Mdm2’s cleavage promotes oridonin‐induced and p53‐mediated NB cells apoptosis and cell cycle arrest. Therefore, we demonstrate that inducing NB cells apoptosis and cell cycle arrest by oridonin is a potential strategy for NB therapy.

## 2 MATERIALS AND METHODS

### 2.1 Chemicals

Oridonin of 98.0% purity was provided by Dr Qingjiu Tang (Shanghai Academy of Agricultural Sciences, China). It was dissolved in DMSO (#67‐68‐5, Aladdin, China) at the concentration of 100 mmol L^−1^ and stored at −20°C. The pan‐caspase inhibitor Z‐VAD‐FMK (#S7023, Selleck, USA) was dissolved in DMSO at the concentration of 50 mmol L^−1^ and stored at −80°C. The antioxidant NAC (N‐Acetyl‐L‐cysteine) (#S0077, Beyotime Biotech, China) was dissolved in ddH_2_O at the concentration of 2 mmol L^−1^ and stored at −20°C. The p53 inhibitor PFT‐α (Pifithrin‐α) (#S2929, Selleck, USA) was dissolved in DMSO at the concentration of 50 mmol L^−1^ and stored at −20°C.

### 2.2 Cell culture

SH‐SY5Y (#SCSP‐5014), SK‐N‐SH (#SCSP‐5029), and SK‐N‐MC (#TCHu 50) cells were kindly provided by Stem Cell Bank (Chinese Academy of Sciences, China). NB41A3 (#CCL‐147, ATCC, USA), 293T (#CRL‐1573, ATCC, USA), HELA (#CCL‐2, ATCC, USA), mouse embryonic fibroblast (MEF), and MEF *Trp53*−/− cells were obtained from Dr Wu Ying‐Li (Department of Pathophysiology, Key Laboratory of Cell Differentiation and Apoptosis of National Ministry of Education, Shanghai Jiaotong University, China). NB41A3 cells were cultured in RPMI 1640 (#SH30809.01, HyClone, USA), supplemented with 10% fatal bovine serun (FBS) (#SV30087.02, HyClone, USA), 10^5^ units L^−1^ penicillin (#15070063, gibco, USA), and 100 g L^−1^ streptomycin (#15070063, gibco, USA). SH‐SY5Y cells were cultured in 45% F‐12 (#C11765500BT, gibco, USA) plus 45% MEM (#SH30024.01, HyClone, USA) supplemented with 10% FBS, 10^5^ units L^−1^ penicillin, 100 g L^−1^ streptomycin, and 2‐mmol L^−1^ glutamine (#25030081, gibco, USA). SK‐N‐MC cells were cultured in MEM supplemented with 10% FBS, 10^5^ units L^−1^ penicillin, 100 g L^−1^ streptomycin, and 2‐mmol L^−1^ glutamine. SK‐N‐SH, 293T, HELA, MEF, and MEF *Trp53*−/− cells were cultured in DMEM (#SH30243.01, HyClone, USA) supplemented with 10% FBS, 10^5^ units L^−1^ penicillin, and 100 g L^−1^ streptomycin. The cells were maintained in the incubator containing 5% CO_2_ at 37°C.^27^

### 2.3 Western blot

Cells (70%‐80% cell confluent monolayer in the 6‐cm cell culture dish) were washed with PBS buffer twice and were added with 300‐μL SDS lysis buffer. The SDS lysis buffer was formulated as follows: 50‐mmol L^−1^ Tris‐HCl (pH6.8) (#ST768, Beyotime Biotech, China), 100‐mmol L^−1^ DTT (#ST040, Beyotime Biotech, China), 2% sodium dodecyl sulfate (SDS) (#ST626, Beyotime Biotech, China), 10% glycerol (#G116202, Aladdin, China), and ddH_2_O. The cell lysate was heated at 100°C for 5 minutes and then was placed on ice for 5 minutes for three times. After centrifuge at 12,000*g* for 1 minute at room temperature, the supernatant of the cell lysate was collected by discarding the pellet. The protein concentration of the cell lysate was determined by the spectrophotometer (#NanoDrop 2000/2000c, Thermo Fisher Scientific, USA). Bromophenol blue (#B8120, Solarbio, China) was added to the cell lysate to the final concentration of 0.05%. The initial loading of the total protein per well was 100 μg, which was adjusted according to the results. The gel consisting of the 10% separation gel and the 5% stacking gel was used to separate proteins with the molecular weight of 30‐120 kDa. The gel consisting of the 12% separation gel and the 5% stacking gel was used to separate proteins with the molecular weight of 10‐30 kDa. Following that, the proteins of the cell lysate were separated via SDS‐PAGE and then were transferred to the nitrocellulose membrane with pore size of 0.22 μm using the wet transfer method. The membrane containing the proteins was transferred to 5% skim milk buffer which was formulated by dissolving the nonfat powdered milk (#A600669, Sangon Biotech, China) in the TBST buffer. The blocking membrane was rocked on shaker for 1 hour at room temperature. And the membrane was further transferred to the buffer containing specific primary antibodies and was rocked on shaker at 4°C overnight. After that, the membrane was washed with the TBST buffer for 5 minutes for three times. Then the membrane was transferred to the buffer containing specific secondary antibodies and was rocked on shaker for 1 hour at room temperature.^28^ Antibodies used were as follows: anti‐cleaved caspase‐3 (#9661, CST, USA, 1:1000, 17/19 kDa), anti‐cleaved caspase‐3 (#9664, CST, USA, 1:1000, 17/19 kDa), anti‐Flag (#14793, CST, USA, 1:1000), anti‐p53 (#2524, CST, USA, 1:1000, 53 kDa), anti‐ubiquitin (#3933, CST, USA 1:1000), anti‐cyclin‐B1 (G2/mitotic‐specific cyclin‐B1) (#4138, CST, USA, 1:1000, 55 kDa), anti‐phospho‐histone H3 (Ser10) (#53348, CST, USA, 1:1000, 17 kDa), anti‐cleaved PARP (poly [ADP‐ribose] polymerase) (#5625, CST, USA, 1:1000, 89 kDa), anti‐GAPDH (glyceraldehyde‐3‐phosphate dehydrogenase) (#5174, CST, USA, 1:5000, 37 kDa), anti‐Mdm2 (#sc‐812, Santa Cruz, USA, 1:250, 90 kDa), anti‐Mdm2‐p60 (#sc‐5304, Santa Cruz, USA, 1:250, 60 kDa), anti‐p53 (#sc‐6243, Santa Cruz, USA, 1:1000, 53 kDa), anti‐BAX (apoptosis regulator BAX) (#sc‐7480, Santa Cruz, USA, 1:1000, 23 kDa), anti‐CDKN1A (cyclin‐dependent kinase inhibitor 1A) (#SAB4500065, Sigma, USA, 1:500, 21 kDa), HRP‐conjugated rabbit anti‐mouse antibody (#315‐035‐003, Jackson Immuno Research, USA, 1:5000), and HRP‐conjugated goat anti‐rabbit antibody (#111‐035‐003, Jackson Immuno Research, USA, 1:5000). The primary antibodies were diluted with the primary antibody dilution buffer (#P0023A, Beyotime Biotech, China). The secondary antibodies were diluted with the 5% skim milk buffer. The membrane was washed again according to the above condition before imaging. ECL reagent (#WBKLS0500, Millipore, USA) was prepared according to the instruction and was added to the membrane. The digital imager (#FUSION FX7, Vilber‐Lourmat, France) with the automatic mode was used for detection. With the GAPDH protein band as a loading control, the gray value of the protein brand was quantitatively analyzed by ImageJ (version k 1.45) software. All experiments were performed three times independently with similar results.

### 2.4 Immunoprecipitation

Cells (70%‐80% cell confluent monolayer in the 6‐cm cell culture dish) were washed with PBS buffer twice and were added with 300‐μL RIPA lysis buffer (#P0013D, Beyotime Biotech, China) containing inhibitors of protease and phosphatase (#4693132001, #490683700, Roche, Switzerland). Then the cell lysate was placed on ice for 30 minutes, and was vibrated every 10 minutes. Following that, the cell lysate was treated by the ultrasonic cell disruptor (power: 200 W; mode: work 4 seconds, stop 3 seconds, for five times) (#DH92‐IIN, LAWSON, China). The supernatant of the cell lysate was collected and divided into two parts: one (250 μL) for the immunoprecipitation assay of the target protein, and the other (30 μL) for the total protein analysis. The protocol for the immunoprecipitation assay was as follows: Two copies of ProteinA + G Agarose Beads (20 μL copy^−1^) (#P2012, Beyotime Biotech, China) were taken for each sample of cell lysate, one for the preincubation of the sample and the other for the preincubation of the antibody. Each copy of ProteinA + G Agarose Beads was washed with RIPA lysis buffer. The sample preincubation: the sample was added with one copy of the washed beads and was inverted at 4°C for 1 hour; the supernatant was collected after centrifuge at 2000*g* for 2 minutes at 4°C. The antibody preincubation: antibody was diluted with the 1‐mL RIPA lysis buffer and was added with another copy of the washed beads, and then was inverted at 4°C for 4 hours; the supernatant was discarded to obtain the beads precipitate after centrifuge at 2000*g* for 2 minutes at 4°C. Antibodies used were as follows: anti‐p53 (#2524, CST, USA, 1:200) and anti‐IgG (#5415, CST, USA). The pretreated sample supernatant and the pretreated antibody binding to the precipitated beads were mixed and inverted at 4°C overnight. Following that, the beads were washed with RIPA lysis buffer five times and the protein binding to the beads was eluted by 30‐μL SDS lysis buffer. After that, the Western Blot protocol was performed. All experiments were performed three times independently with similar results.

### 2.5 Reverse transcription quantitative polymerase chain reaction

Total cellular RNA was extracted from cells using RNAiso Plus reagent (#9109, TaKaRa, Japan) according to the instruction. Reverse transcription PCR was carried out by the PrimeScript RT reagent Kit (#RR047A, TaKaRa, Japan) in a volume of 10 μL for each sample, including 500 ng of total RNA without genomic DNA, 0.5‐μL PrimeScript RT Enzyme Mix, 2‐μL RT Primer Mix, 2‐μL 5 × PrimeScript Buffer, and RNase Free ddH_2_O. The RT Primer Mix of the Kit contains Oligo dT Primer and Random 6 mers. The reaction mixture was initially heated at 37°C for 30 minutes, 85°C for 5 seconds, and finally at 4°C for 5 minutes to get the cDNA. For the quantitative PCR, PCR products were amplified from cDNA using the specific primers and 2 × SYBR Premix Ex Taq II (Tli RNaseH Plus) Kit (#RR820A, TaKaRa, Japan) in the PCR machine (#LightCycler480, Roche, Switzerland). Quantitative PCR was carried out in a volume of 10 μL for each sample, including 5‐μL 2 × SYBR Premix Ex Taq II (Tli RNaseH Plus), 0.4‐μL forward primers (10 μmol L^−1^), 0.4‐μL reverse primers (10 μmol L^−1^), 1‐μL cDNA (<100 ng), and 3.2‐μL RNase Free ddH_2_O. The condition of the two‐step reaction was as follows: pre‐denaturation at 95°C for 30 seconds, denaturation at 95°C for 5 seconds (40 cycles), and annealing/extension at 60°C for 30 seconds. The dissociation curve of each amplification reaction was checked to confirm that there was no nonspecific PCR product. The Ct values of *Trp53 (transformation related protein 53), Actb (actin, beta), TP53, MDM2 (MDM2 proto‐oncogene), and ACTB (actin beta)* were normally 19‐22, 14‐16, 20‐23, 20‐23, and 14‐17, respectively. Relative levels of *Trp53, TP53,* and *MDM2* mRNA were determined by the 2^−△△CT^ method using *Actb or ACTB* as the endogenous control. Primers (Sangon Biotech, China) were synthesized as follows: All experiments were performed three times independently with similar results.

**Table:** 

Target gene	Primer	Nucleotide sequence
*Trp53* (Mouse)	F	5′‐TCACAGCGTCTGTTGACATTT‐3′
	R	5′‐ACCAAGCTCATTACCCTGACA‐3′
*Actb* (Mouse)	F	5′‐CTACCTCATGAAGATCCTGACC‐3′
	R	5′‐CACAGCTTCTCTTTGATGTCAC‐3′
*TP53* (Human)	F	5′‐CAGCACATGACGGAGGTTGT‐3′
	R	5′‐TCATCCAAATACTCCACACGC‐3′
*MDM2* (Human)	F	5′‐AGCTTGAAGCAGTTGGGAGC‐3′
	R	5′‐ACGATCACTTAGGCCAGGCT‐3′
*ACTB* (Human)	F	5′‐ATAGCACAGCCTGGATAGCAACGTAC‐3′
	R	5′‐CACCTTCTACAATGAGCTGCGTGTG‐3′

### 2.6 Plasmids construction and transfection

The pcDNA3.1‐*MDM2* plasmids (#V790‐20, invitrogen) and the pSIN4‐EF1alpha‐IRES plasmids (Vector backbone same as #61062, addgene) were obtained from Dr Ying‐Li Wu. The pcDNA3.1‐*MDM2* (gene ID: 4193) plasmids containing the coding sequence of Mdm2 can be constructed on the basis of pcDNA3.1 plasmids. The coding sequence of Mdm2‐p60 was amplified by PCR using the pcDNA3.1‐*MDM2* plasmids as the template, and the PrimeSTAR GXL DNA Polymerase Kit was used (#R050Q, TaKaRa, Japan). The primers for the PCR were as follows: F: 5′‐GGGCTAGCTAGCTAGGAATTCATGGACTACAAAGACGATGACGACAAGGACTACAAAGACGATGACGACAAGGACTACAAAGACGATGACGACAAGATGGTGAGGAGCAGGCAAAT‐3′; R: 5′‐GCCCTAGATGCATGCGGATCCCTAATCAGGAACATCAAAGCCC‐3′. PCR was carried out in a volume of 50 μL, including 0.5‐μg pcDNA3.1‐*MDM2* plasmids, 2‐μL forward primers (10 μmol L^−1^), 2‐μL reverse primers (10 μmol L^−1^), 4‐μL dNTP Mixture, 10‐μL 5 × PrimeSTAR GXL Buffer (Mg^2+^ plus), 2‐μL PrimeSTAR GXL DNA Polymerase (1.25 U μL^−1^), and RNase Free ddH_2_O. The three‐step reaction condition was as follows: pre‐denaturation at 98°C for 60 seconds, denaturation at 98°C for 10 seconds (30 cycles), annealing at 55°C for 15 seconds, and extension at 68°C for 90 seconds. The PCR products were detected by 1% agarose gel electrophoresis and were purified by the TaKaRa MiniBEST Agarose Gel DNA Extraction Kit Ver.4.0 (#9762, TaKaRa, Japan). The pSIN plasmids were digested by the restriction enzymes BamH I (#1605, TaKaRa, Japan) and EcoR I (#1611, TaKaRa, Japan). The reaction was carried out in a volume of 50 μL, including 1‐μg pSIN plasmids, 1‐μL BamH I, 1‐μL EcoR I, 5‐μL 10 × QuickCut Buffer, and RNase Free ddH_2_O. The reaction condition was as follows: digestion at 37°C for 30 minutes. The reaction was stopped by 0.8% agarose gel electrophoresis, and the linearized pSIN plasmids were detected and purified by the TaKaRa MiniBEST Agarose Gel DNA Extraction Kit Ver.4.0. The ligation reaction of PCR products and the linearized pSIN plasmids was catalyzed by the ClonExpress II One Step Cloning Kit (#C112‐01, Vazyme, China) to obtain the recombinant plasmids coding Mdm2‐p60. The reaction was carried out in a volume of 20 μL, including 160‐ng linearized pSIN plasmids, 60‐ng PCR products, 2‐μL Exnase II, 4‐μL 5 × CE II Buffer, and RNase Free ddH_2_O. The reaction condition was as follows: ligation at 37°C for 30 minutes and on ice for 5 minutes. The recombinant plasmids were amplified and verified by DNA sequencing (GENEWIZ, China).

Cells (50%‐60% cell confluent monolayer in the one well of 6‐well plate) were transfected with pSIN or pSIN‐*Flag3‐MDM2‐p60* plasmids by the Lipofectamine 3000 Transfection Kit (#L3000‐015, Invitrogen, USA). The procedure was as follows: The Mix I solution consists of 6‐μL Lipofectamine 3000 Reagent and 250‐μL Opti‐MEM Medium (#31985‐062, gibco). The Mix II solution consists of 2‐μg pSIN/pSIN‐*Flag3‐MDM2‐p60* plasmids, 4‐μL P3000 Reagent, and 250‐μL Opti‐MEM Medium. The above I and II solutions were thoroughly mixed and placed at room temperature for 5 minutes. Then the mixture was added to one well of 6‐well plate which contains 1.5‐mL normal medium. The transfected cells can be used in the subsequent experiments. All experiments were performed three times independently with similar results.

### 2.7 Cell proliferation assay

The antiproliferative effect of oridonin on cells was assessed by the Cell Count Kit‐8 (#CK04‐3000T, Dojindo, Japan) according to the instruction. Cells were digested by 0.25% trypsin‐EDTA and were washed by PBS buffer twice. Then the cells were added into each well of 96‐well plates and the cell density was 5000 well^−1^. After the cells were attached to the wall, they were treated with 100‐μL medium containing different concentration of oridonin for 24 hours. Then the medium containing oridonin was discarded and 100‐μL new medium containing 10% CCK‐8 reagent was added into each well. The 96‐well plates were placed in the incubator containing 5% CO_2_ at 37°C for 1‐4 hours. The optical density (OD) value of the control group without oridonin treatment should be controlled to 0.8‐1.2, and that can make the results more reliable. The absorbance of each well was measured at 450 nm using a microplate reader (#Synergy2, BioTek, USA). Cell viability = (OD value_the experimental group_−Mean of the OD value_the blank group_)/(Mean of the OD value_the control group_−Mean of the OD value_the blank group_) × 100%. Cell inhibitory rate = 100%−Cell viability. All experiments were performed three times independently with similar results.

### 2.8 Annexin V‐FITC/PI staining

Cell apoptosis was evaluated by the Annexin V‐FITC/PI Apoptosis Detection Kit (#40302ES20, YEASEN, China). Cells were digested by 0.25% trypsin without EDTA and were washed by PBS buffer twice. 1‐5 × 10^5^ cells were precipitated and suspended by 100‐μL 1 × Binding Buffer. Cell suspension was mixed with 5‐μL Annexin V‐FITC plus 10‐μL PI Staining Solution, and was placed at room temperature for 10‐15 minutes out of light. Following that, cell suspension was further mixed with 400‐μL 1 × Binding Buffer. Then the cell suspension should be placed on ice out of light and be detected by flow cytometry (#Epics XL, Beckman Coulter, USA) within 1 hour. All experiments were performed three times independently with similar results.

### 2.9 PI staining

Cell cycle distribution was assessed by Cell Cycle and Apoptosis Analysis Kit (#C1052, Beyotime Biotech, China). Cells were digested by 0.25% trypsin without EDTA, and were washed by PBS buffer twice. Cell precipitation was resuspended and fixed by 75% ice‐cold ethanol at −20°C for 2 hours. Following that, cells were washed by PBS buffer twice and were resuspended by 0.5‐mL staining buffer. The cell density of the cell suspension was about 1 × 10^6^ mL^−1^. The cell suspension was further mixed with 25‐μL 20 × PI staining solution plus 10‐μL 50 × RNase A, and was placed at room temperature for 30 minutes out of light. Then the cell suspension should be placed on ice out of light and be detected by flow cytometry within 24 hours. The FlowJo (version 7.6) software was used for cell cycle analysis. All experiments were performed three times independently with similar results.

### 2.10 Murine model and oridonin treatment

A total of 36 BALB/c nude mice (age: 5 weeks; weight: about 22 g; sex: female) were purchased from Shanghai SLAC Laboratory Animal Company Limited (China), and were maintained in a specific pathogen‐free environment. They were randomly assigned to six groups (six mice per group): four groups for xenograft model and two groups for pseudo‐metastatic model. Animal‐related experiments were performed according to the Guide for the Care and Use of Laboratory Animals (NIH Publications No. 80‐23, revised 1996) and were approved by the committee for human treatment of animals at Shanghai Jiao Tong University School of Medicine.

The xenograft models were established by subcutaneous injection of 2 × 10^6^ NB41A3 cells in the right lower abdomen of the mice. When tumor became palpable, the nude mice were administered with drug (PBS, 10% DMSO, 10 or 20 mg kg^−1^ oridonin) everyday by intraperitoneal injection. The longest diameter and its according perpendicular diameter of each tumor were measured everyday using caliper to estimate the tumor volume. The formula followed was used to calculate the volume of the tumor: 4π/3 × (width/2)^2^ × (length/2). All the nude mice were sacrificed at the 28th day, and the tumors were collected for further experiments.

The pseudo‐metastatic models were established by intravenous injection of 2 × 10^5^ NB41A3 cells through the tail vein of the mice. After 3 days, the nude mice were administered with drug (0.2 mL PBS or 20 mg kg^−1^ oridonin) every other day for 2 weeks by intraperitoneal injection. In the experiments, mice were monitored routinely for weight loss and were sacrificed when signs of poor health became evident. Survival time was used as the main criterion for determining treatment efficacy.

### 2.11 Immunohistochemistry

Several sections with the thickness of 5 μm were cut from each paraffin‐embedded tumor. Subsequently, these sections were immersed in xylene twice, 30 minutes each time. And, then they were transferred to graded ethanol. After that, they were completely rinsed by dH_2_O. The sections were then placed in a high‐pressure steamer containing 1 × Citrate Antigen Retrieval Solution (#P0081, Beyotime Biotech, China) to retrieve antigens by boiling for about 2 minutes. Endogenous peroxidase activity was blocked by immersing sections in 3% hydrogen peroxide‐methanol buffer for 5 minutes. The sections were next blocked with 5% BSA‐PBS buffer (#A600332, Sangon Biotech, China) for 40 minutes at room temperature to reduce nonspecific staining. Following that, the sections were transferred to the buffer containing specific primary antibodies at 4°C overnight. After the sections were sufficiently rinsed by PBS buffer, they were transferred to the buffer containing the HRP‐conjugated secondary antibodies for 40 minutes at room temperature. The sections were stained for 5‐10 minutes at room temperature out of light by the DAB Kit (#P0203, Beyotime Biotech, China). Finally, the sections were stained with hematoxylin and eosin. Antibodies used were as follows: anti‐p53 (#sc‐6243, Santa Cruz, USA, 1:100, 53 kDa), anti‐CDKN1A (#SAB4500065, Sigma, USA, 1:100, 21 kDa), HRP‐conjugated rabbit anti‐mouse antibody (#315‐035‐003, Jackson Immuno Research, USA, 1:1000), and HRP‐conjugated goat anti‐rabbit antibody (#111‐035‐003, Jackson Immuno Research, USA, 1:1000). The primary antibodies and secondary antibodies were diluted with 5% BSA‐PBS buffer. The sections were observed and photographed by the inverted microscope (#ECLIPSE Ti‐S, Nikon, Japan) using the NIS‐Elements (BR 4.10.00) software under the 200× magnification. The ImageJ (version k 1.45) software was used for analysis.

### 2.12 TUNEL

For preparing single‐cell suspension, the technique described in a previous report was used.^29^ The cell density of the cell suspension was about 2 × 10^7 ^mL^−1^. About 50‐100 μL cell suspension was put onto each polylysine‐coated section and was gently smeared with a clean section. Cells on sections were immersed in 4% poly‐formaldehyde‐PBS buffer at 4°C for 25 minutes to be fixed, and they were further incubated with 100‐μL Proteinase K‐PBS buffer (20 μg mL^−1^) at room temperature for 40 minutes. Apoptosis of cells of different tumors was measured by TUNEL Apoptosis Detection Kit (Alexa Fluor 488) (#40307ES20, YEASEN, China). The sections were incubated with 100‐μL 1 × Equilibration Buffer for 10‐30 minutes at room temperature. After that, the 1 × Equilibration Buffer was absorbed by absorbent paper. Then the sections were incubated with 50‐μL TdT Enzyme Buffer for 60 minutes at room temperature out of light. Following that, the sections were immersed in PBS buffer for 5 minutes at room temperature, and then were rinsed completely by PBS buffer. The sections were stained with 1 μg mL^−1^ PI‐PBS buffer for 5 minutes, and were also stained again with 2 μg mL^−1^ DAPI‐PBS buffer for 5 minutes. Both staining procedure should be at room temperature out of light. After that, the sections were rinsed completely by dH_2_O. The percentage of apoptotic cells was determined by counting at least 1000 cells in 10 to 20 fields under the 200× magnification using phase‐contrast and fluorescent microscopy.

### 2.13 Statistical analysis

The measurement data are presented as the mean ± SD. The statistical analysis was performed using the software GraphPad Prism 5 or SPSS 22.0. A value of *P* < 0.05 was considered to be statistically significant. Nonparametric tests were used.

Statistical analysis of growth inhibition induced by oridonin on cancer cells, relative expression of mRNA, relative expression of protein, tumor volume, and tumor weight was analyzed with Kruskal‐Wallis test and was presented as “*P*”, when Kruskal‐Wallis test was significant it was followed by Dunn's multiple comparisons test and was presented as “*P_n_*”. Statistical analysis of growth inhibition induced by oridonin on MEF and MEF *Trp53*−/− was analyzed using Mann‐Whitney *U* test and was presented as “*P*”. IC50 value was analyzed by GraphPad Prism 5. Statistical analysis of mice survival time was analyzed using Log‐rank (Mantel‐Cox) test and was presented as “*P*”.

## 3 RESULTS

### 3.1 Oridonin suppressed the proliferation of NB cells by inducing cell apoptosis and cell cycle arrest

**Figure 1:**
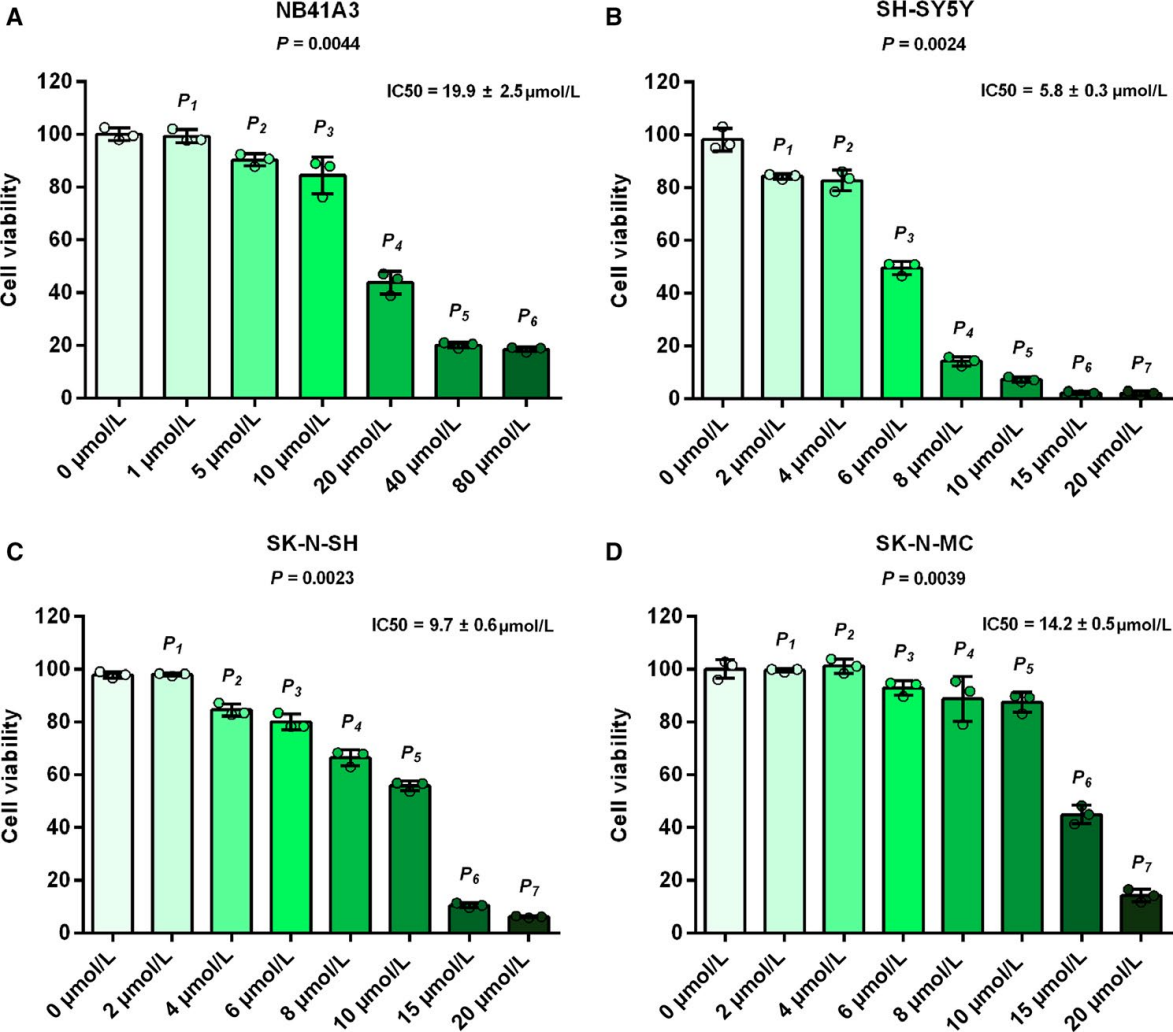
Oridonin inhibited NB cells growth with different concentration. NB cell lines were treated with a series of different concentration of oridonin indicated for 24 hour. Cell viability was determined by the CCK‐8 kit. The dots in the graphs represent independent experimental replicates. (A) NB41A3 cells were treated with 0, 1, 5, 10, 20, 40, and 80 μmol L^−1^ oridonin. *P*_1_ > 0.9999 (1 vs 0 μmol L^−1^), *P*_2_ > 0.9999 (5 vs 0 μmol L^−1^), *P*_3_ > 0.9999 (10 vs 0 μmol L^−1^), *P*_4_ = 0.5770 (20 vs 0 μmol L^−1^), *P*_5_ = 0.0710 (40 vs 0 μmol L^−1^), and *P*_6_ = 0.0240 (80 vs 0 μmol L^−1^). (B) SH‐SY5Y cells were treated with 0, 2, 4, 6, 8, 10, 15, and 20 μmol L^−1^ oridonin. *P*_1_ > 0.9999 (2 vs 0 μmol L^−1^), *P*_2_ > 0.9999 (4 vs 0 μmol L^−1^), *P*_3_ > 0.9999 (6 vs 0 μmol L^−1^), *P*_4_ > 0.9999 (8 vs 0 μmol L^−1^), *P*_5_ = 0.2620 (10 vs 0 μmol L^−1^), *P*_6_ = 0.0200 (15 vs 0 μmol L^−1^), and *P*_7_ = 0.0200 (20 vs 0 μmol L^−1^). (C) SK‐N‐SH cells were treated with 0, 2, 4, 6, 8, 10, 15, and 20 μmol L^−1^ oridonin. *P*_1_ > 0.9999 (2 vs 0 μmol L^−1^), *P*_2_ > 0.9999 (4 vs 0 μmol L^−1^), *P*_3_  > 0.9999 (6 vs 0 μmol L^−1^), *P*_4_  > 0.9999 (8 vs 0 μmol L^−1^), *P*_5_ = 0.5850 (10 vs 0 μmol L^−1^), *P*_6_ = 0.1300 (15 vs 0 μmol L^−1^), and *P*_7_ = 0.0230 (20 vs 0 μmol L^−1^). (D) SK‐N‐MC cells were treated with 0, 2, 4, 6, 8, 10, 15, and 20 μmol L^−1^ oridonin. *P*_1_ > 0.9999 (2 vs 0 μmol L^−1^), *P*_2_ > 0.9999 (4 vs 0 μmol L^−1^), *P*_3_ > 0.9999 (6 vs 0 μmol L^−1^), *P*_4_ > 0.9999 (8 vs 0 μmol L^−1^), *P*_5_ > 0.9999 (10 vs 0 μmol L^−1^), *P*_6_ = 0.2210 (15 vs 0 μmol L^−1^), and *P*_7_ = 0.0420 (20 vs 0 μmol L^−1^)

**Figure 2:**
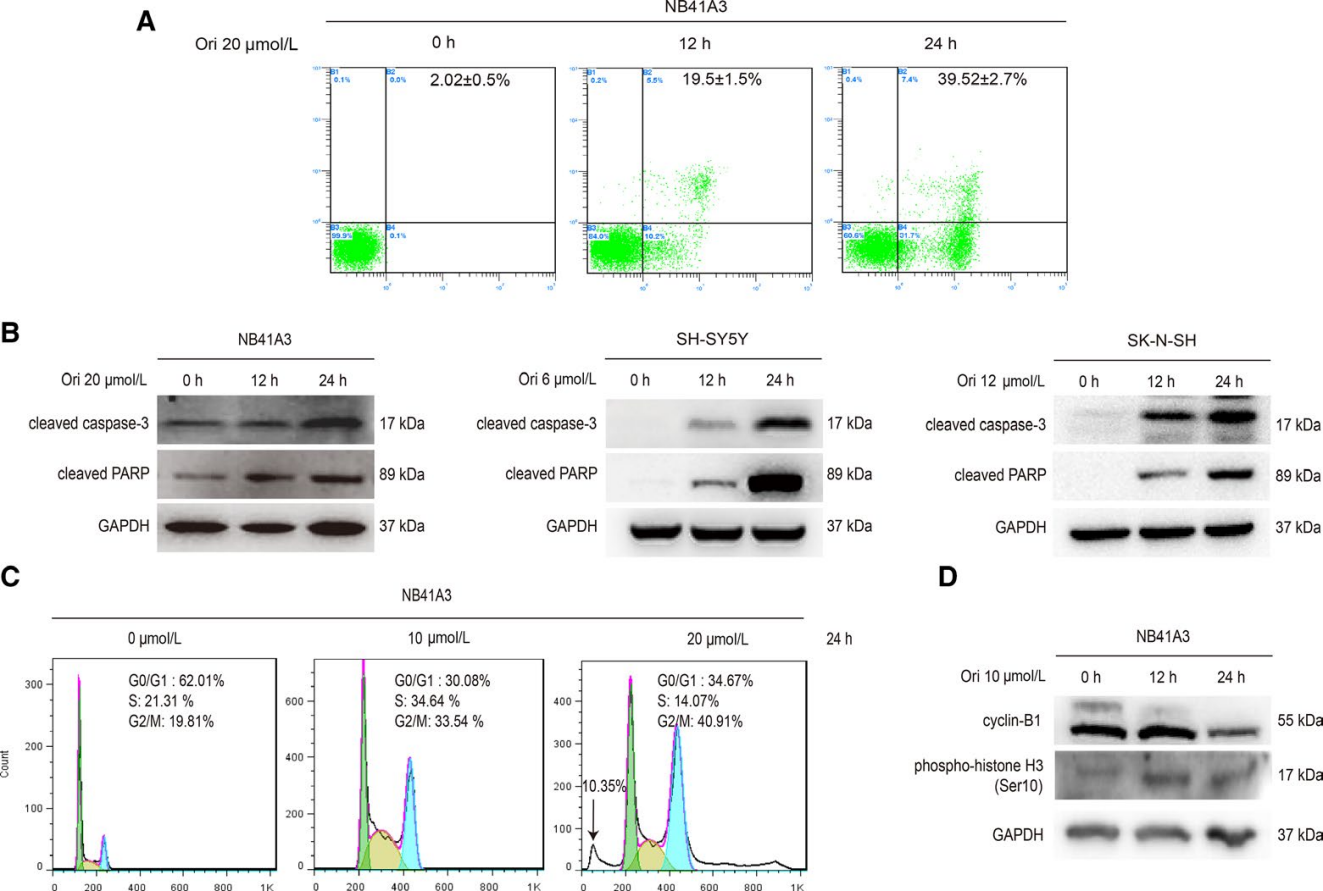
Oridonin suppressed the proliferation of NB cells by inducing cell apoptosis and cell cycle arrest. (A) NB41A3 cells were treated with 20‐μmol L^−1^ oridonin for 0, 12, and 24 hour, respectively, and then cells were collected and apoptosis of cells was detected by flow cytometry. (B) NB41A3, SH‐SY5Y, and SK‐N‐SH cells were treated with 20, 6, and 12 μmol L^−1^ oridonin, respectively, for the indicated time. The expression of cleaved caspase‐3 and cleaved PARP was determined by Western Blot analysis. (C) NB41A3 cells were treated with 0, 10, and 20 μmol L^−1^ oridonin, respectively, for 24 hour, and then cells were collected and cell cycle distribution was detected by flow cytometry. (D) NB41A3 cells were treated with 10‐μmol L^−1^ oridonin for 0, 12, and 24 hour, respectively, and then the expression of cyclin‐B1 and phospho‐histone H3 (Ser10) was assessed by Western Blot analysis

We initially treated the NB41A3, SH‐SY5Y, SK‐N‐SH, and SK‐N‐MC cells with a series of different concentration of oridonin for 24 hours, and observed that oridonin could suppress the proliferation of NB cells (Figure 1A‐D). To identify the mechanisms of the oridonin's inhibitory effects on NB cells, NB41A3 cells were treated with 20‐μmol L^−1^ oridonin for 12 and 24 hours. Then, cell necrosis and apoptosis were measured by Annexin V/PI staining using flow cytometry. The data proved that oridonin could induce NB41A3 cells apoptosis in the time‐dependent manner (Figure 2A). For the detailed effects of oridonin on NB cells, NB41A3 cells were also treated with 10‐ or 20‐μmol L^−1^ oridonin for 24 hours. Cell cycle percentage detected by flow cytometry analysis showed that oridonin induced NB41A3 cells to be arrested in G2/M phase (Figure 2C). Furthermore, 20‐μmol L^−1^ oridonin also caused a significant increase in SubG1 phase (Figure 2C). Additionally, the oridonin‐induced expression of cleaved caspase‐3 and cleaved PARP in different NB cell lines further confirmed that oridonin could induce apoptosis (Figure 2B). After NB41A3 cells treated with 10‐μmol L^−1^ oridonin for 12 and 24 hours, the expression of cyclin‐B1 and phospho‐histone H3 (Ser10) was also enhanced (Figure 2D). These results indicated that oridonin may suppress the proliferation of NB cells by inducing cell apoptosis and cell cycle arrest.

### 3.2 Oridonin‐induced p53 accumulation led NB cells apoptosis and cell cycle arrest

**Figure 3:**
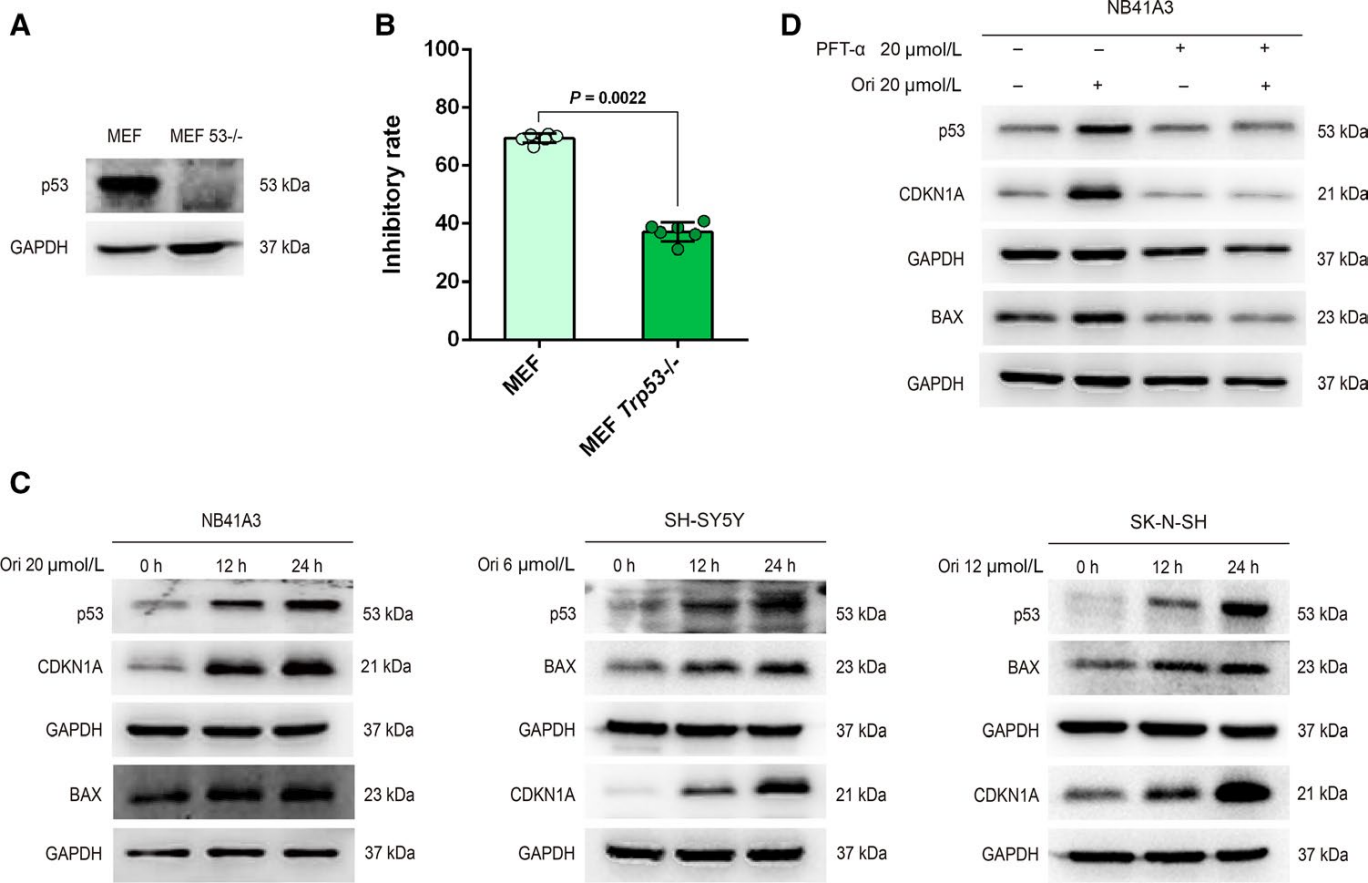
Oridonin‐induced p53 accumulation led NB cells apoptosis and cell cycle arrest. (A‐B) MEF and MEF *Trp53*−/− cells were treated with 20‐μmol L^−1^ oridonin for 24 hours. The inhibitory effects were detected by the CCK‐8 kit. The dots in the graphs represent independent experimental replicates. (C) NB41A3, SH‐SY5Y, and SK‐N‐SH cells were treated with 20, 6, and 12 μmol L^−1^ oridonin, respectively, for the indicated time. The expression of p53, CDKN1A, and BAX was assessed by Western Blot analysis. (D) NB41A3 cells were pretreated with DMSO or 20‐μmol L^−1^ PFT‐α for 24 hours. In the presence of DMSO or PFT‐α, NB41A3 cells were treated with DMSO or 20‐μmol L^−1^ oridonin for another 12 hours. The expression of p53, CDKN1A, and BAX was detected by Western Blot analysis

Based on the previous studies, we carried out research on p53. The MEF and MEF *Trp53*−/− cells were treated with 20‐μmol L^−1^ oridonin for 24 hours (Figure 3A). The difference of inhibitory effects suggested that p53 may play a role in the cell growth inhibition induced by oridonin (Figure 3B). We then detected the effects of oridonin on the expression of p53. As shown in the results, oridonin indeed increased the expression of p53 in the NB cell lines (Figure 3C). And, it was also found that the expression of CDKN1A and BAX was increased (Figure 3C), they are proteins regulated by p53 and are involved in the apoptosis and cell cycle arrest. These results were consistent with the previous theory that p53 accumulation can lead to cell cycle arrest and apoptosis by regulating the transcription of its target gene.^30^ To further validate p53’s role in the oridonin‐induced apoptosis and cell cycle arrest, we pretreated NB41A3 cells with the p53 inhibitor PFT‐α and observed that the upregulation of CDKN1A and BAX induced by oridonin was totally reversed by PFT‐α (Figure 3D). These results indicated that oridonin can induce NB cells apoptosis and cell cycle arrest by reactivating p53 and regulating the proteins on the downstream of p53.

### 3.3 Mdm2‐p60 was generated in the apoptosis process to maintain p53’s continuous activation

**Figure 4:**
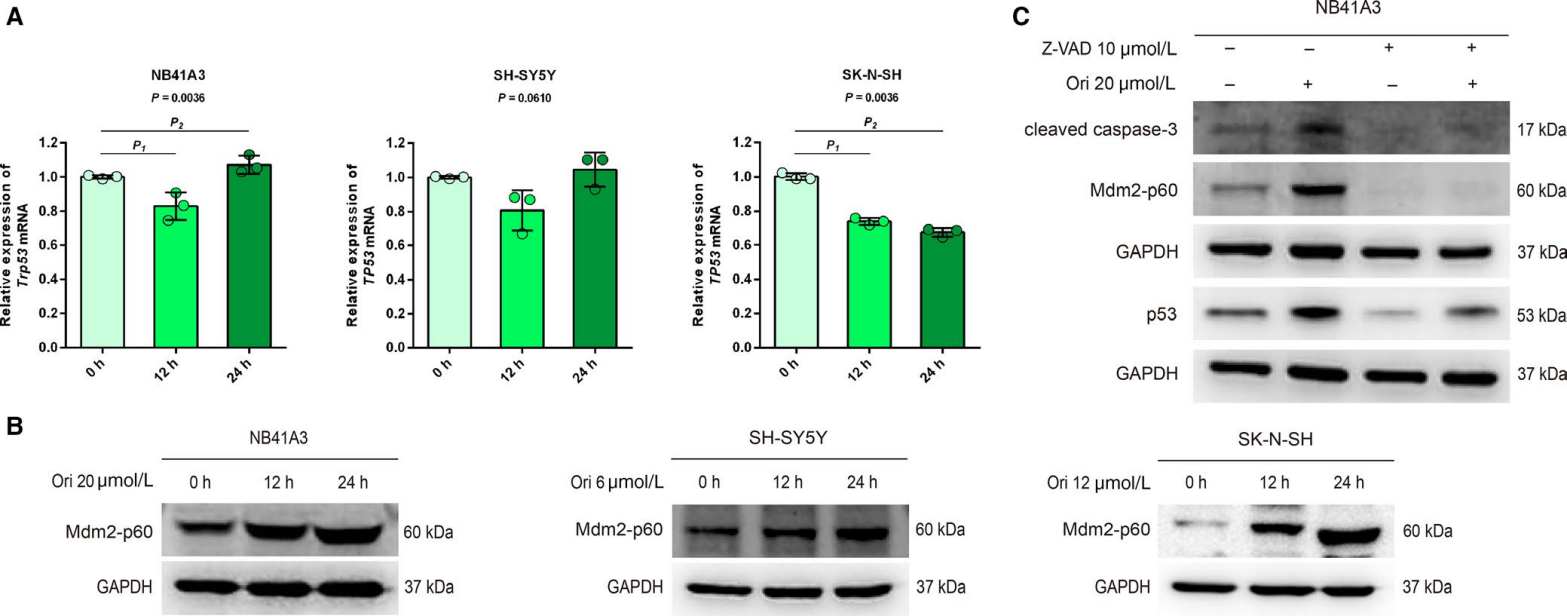
Mdm2‐p60 was generated in the apoptosis process to maintain p53’s continuous activation. (A) NB41A3, SH‐SY5Y, and SK‐N‐SH cells were treated with 20, 6, and 12 μmol L^−1^ oridonin, respectively, for the indicated time. *Trp53* and *TP53* mRNA levels were determined by real‐time RT‐PCR. The dots in the graphs represent independent experimental replicates. For NB41A3 cells, *P*_1_ = 0.5390 (12 vs 0 hour), *P*_2_ = 0.5390 (24 vs 0 hour). For SK‐N‐SH cells, *P*_1_ = 0.5340 (12 vs 0 hours), *P*_2_ = 0.0210 (24 vs 0 hour). (B) NB41A3, SH‐SY5Y and SK‐N‐SH cells were treated with 20, 6, and 12 μmol L^−1^ oridonin, respectively, for the indicated time. The expression of Mdm2‐p60 was detected by Western Blot analysis. (C) NB41A3 cells were pretreated with DMSO or 10‐μmol L^−1^ Z‐VAD for 1 hour. In the presence of DMSO or Z‐VAD, NB41A3 cells were treated with 20‐μmol L^−1^ oridonin or DMSO, respectively, for 12 hours. The expression of cleaved caspase‐3, Mdm2‐p60, and p53 was detected by Western Blot analysis

There was no significant change in the mRNA level of *TP53* when the NB cells were treated with oridonin (Figure 4A). Mdm2, which acts as the E3 ubiquitin ligase for p53, is considered to be the major negative regulator of p53,^31^ and the transcription of *MDM2* also can be trans‐activated by p53. Under oridonin treatment, the expression of Mdm2 was increased probably because *MDM2*’s transcription was enhanced by p53 (Figure S1A and B). Moreover, it could also be observed in all NB cell lines that oridonin induced the cleavage of Mdm2 and the generation of Mdm2‐p60 (Figure 6B). Proteolytic cleavage of the Mdm2 was reported to depend on the activation of specific caspases.

Previous studies have proved that Mdm2‐p60 failed to target p53 for degradation.^32^ Contrarily, Mdm2‐p60 can bind to p53 to improve the p53’s stability. In support of this view, when the induction of Mdm2‐p60 was blocked by caspase inhibitor Z‐VAD, the accumulation of p53 induced by oridonin was greatly decreased (Figure 4C). Taken together, these results indicated that oridonin‐induced Mdm2‐p60 can maintain the continuous activation of p53.

### 3.4 Mdm2‐p60 improved the p53’s stability by reducing its ubiquitination

**Figure 5:**
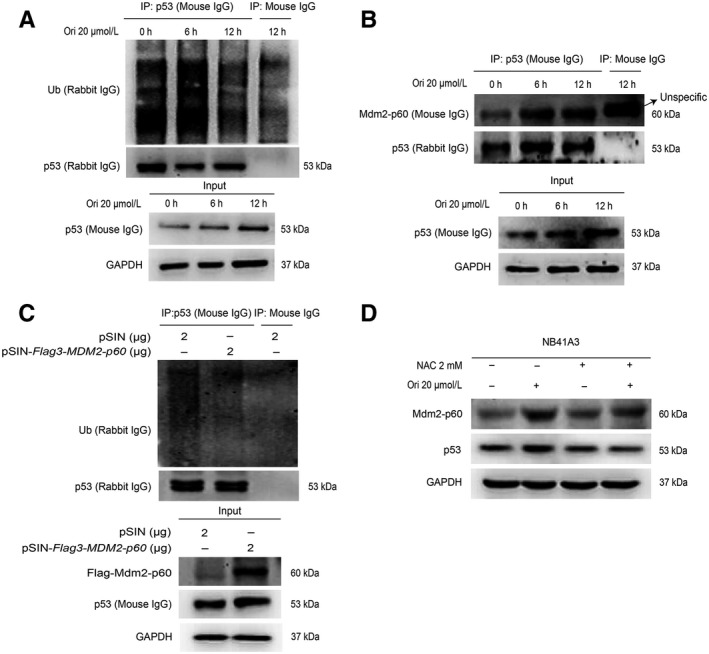
Mdm2‐p60 improved the p53’s stability by reducing its ubiquitination. NB41A3 cells were treated with 20‐μmol L^−1^ oridonin for 0, 6, and 12 hours, respectively. Cell lysate was subjected to immunoprecipitation analysis to detect the ubiquitination of p53 (A) and the binding of p53 to Mdm2‐p60 (B). (C) pSIN or pSIN‐*Flag3‐MDM2‐p60* plasmids were transfected into NB41A3 cells, and 48 hours after transfection cells were treated with 20‐μmol L^−1^ MG132 for 0.5 hour. Then, p53 was immunoprecipitated from the cell lysate, and the ubiquitination of p53 was assessed by Western Blot analysis. (D) NB41A3 cells were pretreated with PBS or 2‐mmol L^−1^ NAC for 1 hour. In the presence of PBS or NAC, NB41A3 cells were treated with DMSO or 20‐μmol L^−1^ oridonin for 12 hours. The expression of Mdm2‐p60 and p53 was assessed by Western Blot analysis

Our results showed that oridonin may induce the accumulation of p53 by decreasing the ubiquitination of p53 (Figure 5A). We also discovered that Mdm2‐p60 bound with p53, and p53’s ubiquitination reduction induced by Mdm2‐p60 may result in the improvement of p53’s stability (Figure 5B and 5C). Furthermore, it was also demonstrated that the increased expression of Mdm2‐p60 and p53 was also associated with ROS (Figure 5D).

### 3.5 Oridonin inhibited the growth of NB xenograft with p53 and CDKN1A upregulation

**Figure 6:**
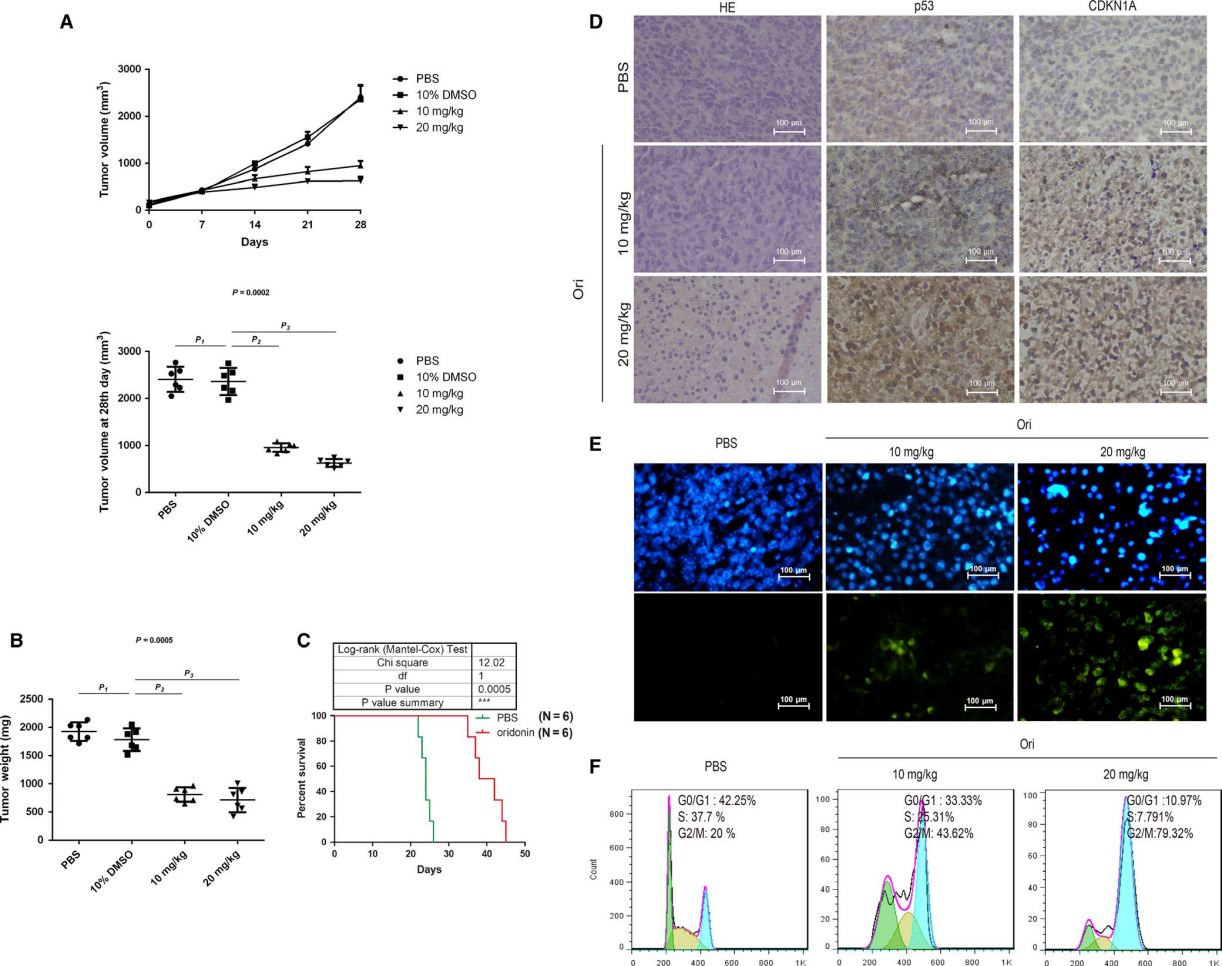
Oridonin inhibited the growth of NB xenograft with p53 and CDKN1A upregulation. NB41A3 cells (2 × 10^6^) were subcutaneously injected into nude mice. When tumors became palpable, the nude mice were administered with drug (PBS, 10% DMSO, 10 or 20 mg kg^−1^ oridonin) everyday by intraperitoneal injection. (A) The sizes of tumor were monitored at the time indicated. The dots in the graphs represent independent biological replicates. *P*_1_ > 0.9999 (10% DMSO vs PBS), *P*_2_ = 0.2470 (10% DMSO vs 10 mg kg^−1^), and *P*_3_ = 0.0030 (10% DMSO vs 20 mg kg^−1^). (B) The weights of tumor were monitored at the time indicated. The dots in the graphs represent independent biological replicates. *P*_1_ > 0.9999 (10% DMSO vs PBS), *P*_2_ = 0.0960 (10% DMSO vs 10 mg kg^−1^), and *P*_3_ = 0.0220 (10% DMSO vs 20 mg kg^−1^). (C) The pseudo‐metastatic models were established by intravenous injection of NB41A3 cells through the tail vein of the mice. After 3 days, the mice were administered with 0.2 mL of PBS or oridonin (20 mg kg^−1^) by intraperitoneal injection every other day for 2 weeks. (D) Expression of p53 and CDKN1A in tumor tissues of the indicated group by immunohistochemistry. (Bar = 100 μm) (E) Apoptosis of tumor cells of the indicated group by TUNEL assay. (Bar = 100 μm) (F) Cell cycle distribution of tumor cells of the indicated group by flow cytometry analysis

The xenograft models were administered with drug (PBS, 10% DMSO, 10 or 20 mg kg^−1^ oridonin) everyday by intraperitoneal injection for 28 days. Oridonin treatment resulted in the suppression of xenograft growth, compared with the DMSO treatment (Figure 6A). It was also observed that the treatment of oridonin could reduce the tumor weight, compared with the DMSO treatment (Figure 6B). The pseudo‐metastatic models were established by intravenous injection of NB41A3 cells through the tail vein of the mice. After 3 days, the mice were administered with drug (0.2‐mL PBS or 20 mg kg^−1^ oridonin) every other day for 2 weeks by intraperitoneal injection. The results showed that oridonin treatment prolonged the lifespan of the pseudo‐metastatic models, compared with the PBS treatment (Figure 6C). By H&E staining, sparse cancer cells and areas of necrosis can be seen in the tumor tissue of the oridonin treatment group, while massive cancer cells were observed in the tumor tissue of the PBS treatment group (Figure 6D). By immunohistochemistry, we found that the expression of p53 and CDKN1A was increased (Figure 6D).

To further confirm the effects of oridonin on cancer cells in vivo, single‐cell suspension was derived from the tumor tissue of different groups. A large number of apoptotic cells could be observed in the oridonin treatment group by TUNEL assay (Figure 6E). Meanwhile, cell cycle percentage was analyzed by PI staining. The data indicated that both the low and high dose of oridonin induced tumor cells to be arrested in G2/M phase (Figure 6F).

## 4 DISCUSSION

**Figure 7:**
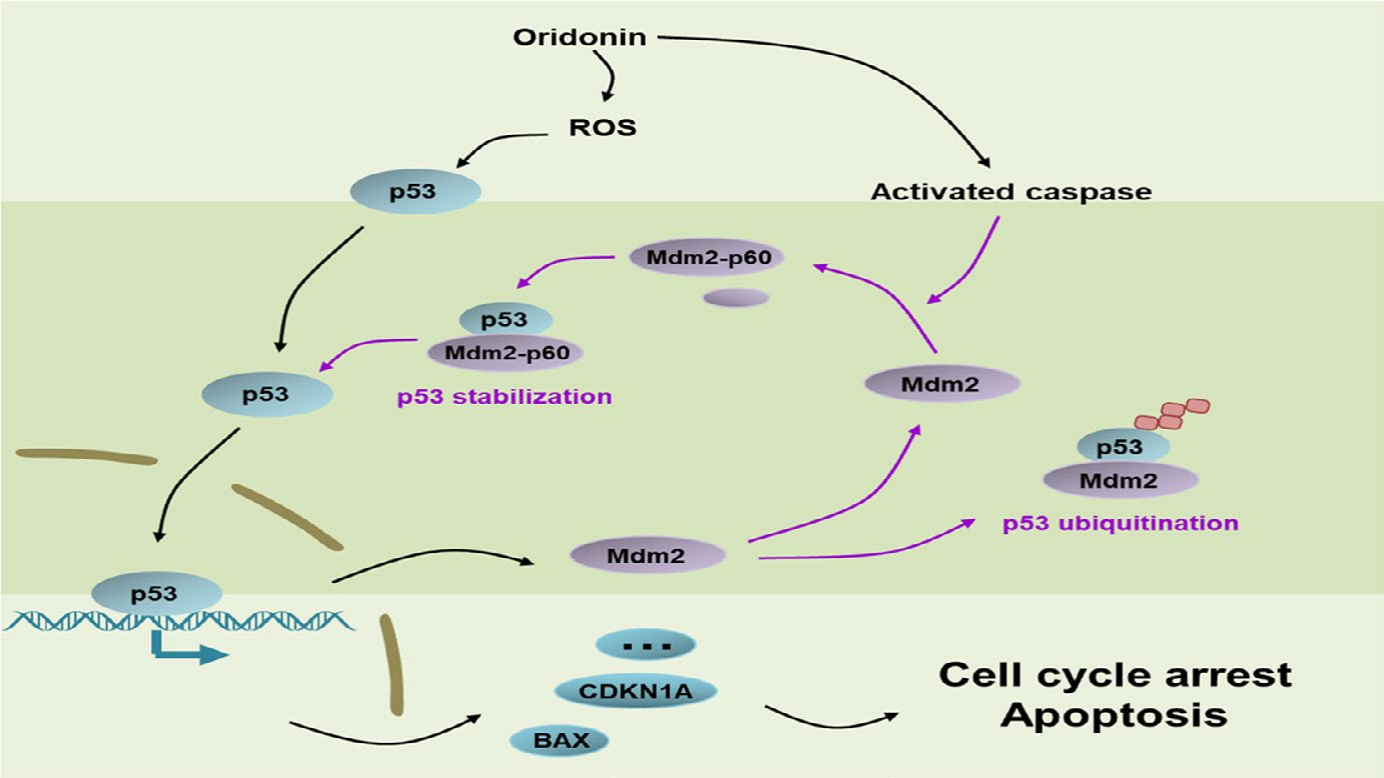
Schematic representation of the mechanisms of oridonin‐induced cell apoptosis and cell cycle arrest in NB. Oridonin‐induced p53 was related with ROS. Oridonin‐induced p53 as a transcription factor regulated the expression of a series of apoptosis‐ and cycle cycle‐related proteins, resulting in apoptosis and cell cycle arrest of NB cells. However, *MDM2*’s transcription was also upregulated by p53. Oridonin induced the activation of caspases to cleave Mdm2 to generate Mdm2‐p60. Mdm2 could act as the E3 ubiquitin ligase of p53 to promote the degradation of p53, while Mdm2‐p60 can stabilize p53 to maintain p53’s continuous activation

The low‐level of p53 in NB cells is an important link in the development and progression of NB. Exploring methods to increase the expression of p53, which can cause cell cycle arrest and apoptosis, thereby playing a role in inhibiting NB cells growth, is a worthy research direction. In our research, we find that oridonin could induce Mdm2‐p60 to promote p53‐mediated apoptosis and cell cycle arrest in NB (Figure 7).

Oridonin was observed to inhibit cell growth and induce cell cycle arrest and apoptosis in NB cells. Since oridonin and its derivatives have been reported to be involved in the activation of p53‐related pathways, we selected *Trp53*‐deficient MEF and NB cells to observe the role of p53 in the oridonin's effects. As expected, the different inhibition effects on MEF and MEF *Trp53*−/− cells, and the expression of p53 and the proteins on the downstream of p53, both proved that oridonin's effects partially depended on p53. And, the rescue of oridonin‐induced CDKN1A and BAX by PFT‐α further confirmed p53’s role in the oridonin's effects.

It was observed that the change of TP53 mRNA level was not the reason for the enhanced expression of p53 in NB cells. And, this finding is inconsistent with a previous report in gastric cancer cells.^22^ This may be due to the fact that cells of different origins respond differently to oridonin: gastric cancer cells originate from epithelial cells and NB cells originate from neural crest. Mdm2 is the major negative regulator of p53, for it can promote the degradation of p53 by increasing its ubiquitination. When NB cells were treated with oridonin, it was observed that Mdm2‐p60, which is a proteolytic cleavage product of Mdm2, was significantly increased. And the generation of Mdm2‐p60 was dependent on caspase‐3 activation.

In fact, Mdm2 consists of 491 amino acids, and it contains a p53 Binding Domain at the N‐terminus and a Ring Domain which is essential for the E3 ubiquitin ligase activity at the C‐terminus.^33^, ^34^ Mdm2 can be cleaved by specific caspases to generate Mdm2‐p60 which is a 60 kDa fragment containing 1‐361 amino acids of Mdm2; moreover, the conserved sequence DVPD (358‐361 amino acids) of Mdm2 is the caspase recognition site.^32^, ^34^, ^35^, ^36^ Cooper GM et al firstly observed that in apoptotic cells, caspase‐3 cleaved Mdm2 and Mdm2‐p60 were generated.^35^ Chen J et al subsequently found that Mdm2‐p60 existed in non‐apoptotic cancer cells.^36^ Mdm2‐p60 loses the Ring Domain so that it cannot target p53 to proteasome degradation, but with the remaining p53 Binding Domain it still can bind with p53.^32^ Tyler J et al proposed that after the DNA damage of cells, Mdm2‐p60 maintained the p53’s stability by reducing the modifications of p53.^32^ During the oridonin‐induced apoptosis of NB cells, we first discovered that the generated Mdm2‐p60 maintained p53’s continuous activation by reducing p53’s ubiquitination (Figure 5C). Caspase‐2 and caspase‐3 are caspases currently reported to generate Mdm2‐p60,^32^ and previous studies and our results showed that oridonin can activate caspase‐3, which may participate in the oridonin‐induced Mdm2’s cleavage. The transcription of full‐length *MDM2* with 10 coding exons can be regulated by both the P1 and P2 promoter. Meanwhile, p53 can upregulate *MDM2* mRNA by inducing the *MDM2*’s transcription from the P2 promoter. This is probably the reason for the oridonin‐induced upregulation of Mdm2. However, Mdm2 is considered to be the major negative regulator of p53.^31^ For it can bind to p53 to block p53‐mediated transcriptional regulation, and also acts as an E3 ligase to promote the proteasome degradation of p53.^37^, ^38^ According to this, Mdm2’s cleavage induced by caspases seems to be significant for p53’s continuous activation in oridonin‐induced apoptosis and cell cycle arrest. Additionally, oridonin has been proved to be a small inhibitor of nucleolin,^39^ which participates in nucleolar stress associated with specific ribosomal proteins.^40^ Under nucleolar stress, ribosomal proteins‐Mdm2‐p53 pathway can result in p53 stabilization and activation, so it is well worthy to further explore whether oridonin reactivates p53 via regulation on nucleolin and related ribosomal proteins.^41^

Consistent with previous studies in other cancers, oridonin induced NB cells apoptosis and cell cycle arrest by reactivating p53. Although it is worth noting that the reactivation of p53 is an important molecular mechanism of oridonin, our research showed that the effects of oridonin may not be completely dependent on P53. In MEF *Trp53*−/− cells, oridonin reduced cell's viability by approximately 40% (Figure 3B). As well as in HELA cells where p53 is degraded due to the presence of HPV18 E6, oridonin treatment cannot induce significant increase in p53 but increase CDKN1A and BAX significantly (Figure S1D). Similarly, in *TP53* null colon cancer cells, 5‐FU (5‐fluorouracil) can cause nucleolar stress to lead to cell cycle arrest and apoptosis. The nucleolar stress induced by 5‐FU can further activate rpL3 (ribosomal protein L3), which induce the expression of its target gene *CDKN1A* and regulates it's another target *gene CBS (cystathionine‐beta‐synthase)* to induce mitochondrial apoptosis characterized by an increase of BAX.^42^, ^43^ This suggests that it is worth to explore whether oridonin can lead to cell cycle arrest and apoptosis via p53‐independent pathways, such as inducing nucleolar stress to regulate ribosomal proteins like rpL3.

In summary, we identify the oridonin's effects on NB cells and the molecular mechanisms of the effects. Oridonin induces apoptosis and cell cycle arrest of NB cells by reactivating p53. Besides, the cleavage of Mdm2 promotes oridonin‐induced p53‐mediated cell apoptosis and cell cycle arrest. Moreover, there are several reports about oridonin's derivatives which are with higher activity. These data provide clues and directions for the exploration of oridonin's derivatives, from which we hope to find more effective derivatives. Based on these efforts, we propose that oridonin exerts its antitumor activity partially by targeting the Mdm2‐p53 axis in NB cells, and oridonin and its derivatives may be potential drugs for NB treatment.

## CONFLICTS OF INTEREST

The authors confirm that there are no conflicts of interest.
