# Supplementary figures and images for: Oridonin induces Mdm2‐p60 to promote p53‐mediated apoptosis and cell cycle arrest in neuroblastoma
Source: Cancer Med. 2019 Jul 24;8(11):5313–26. doi: 10.1002/cam4.2393 (PMC6718599; doi:10.1002/cam4.2393)

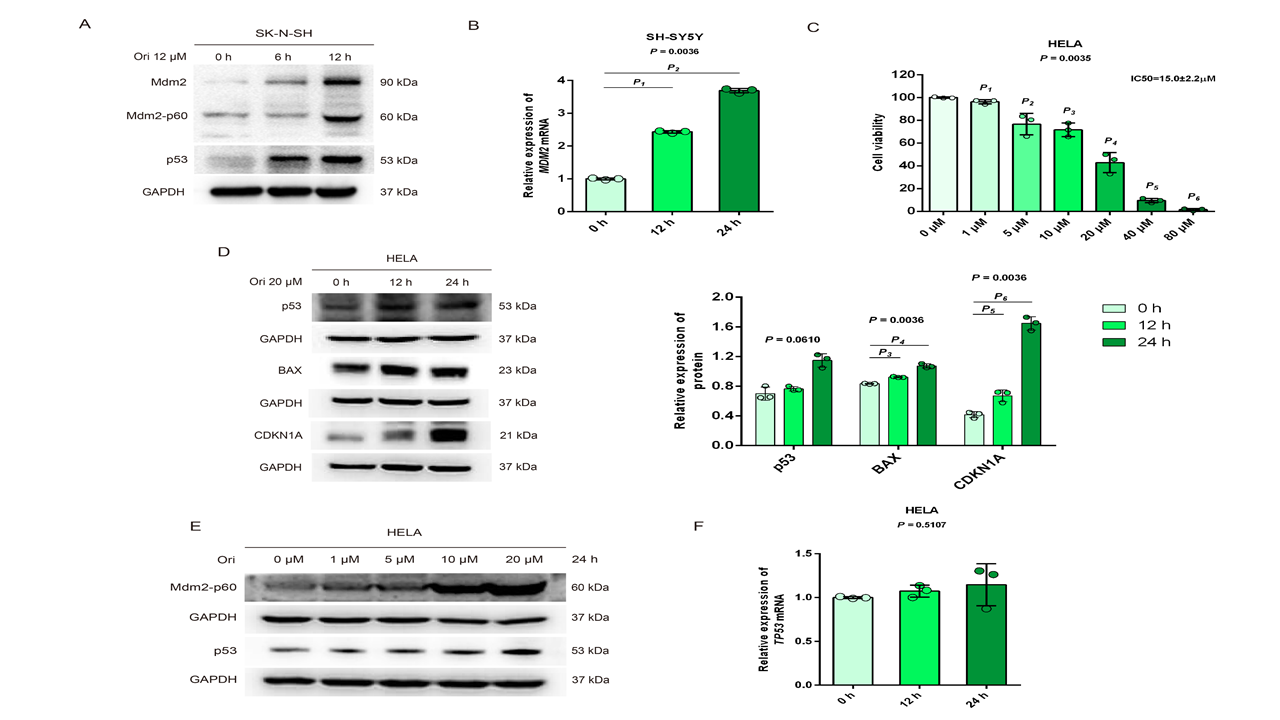

Supplement: Supplementary file 1 [file CAM4-8-5313-s001.tif]

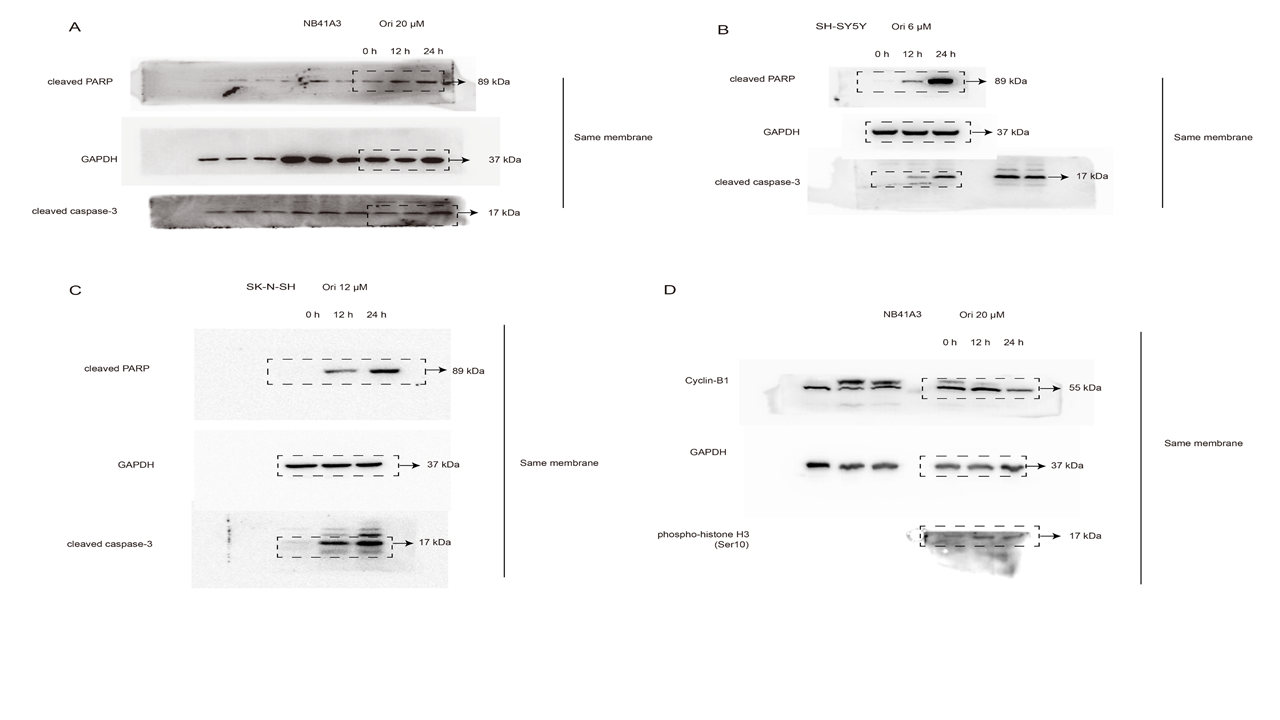

Supplement: Supplementary file 2 [file CAM4-8-5313-s002.tif]

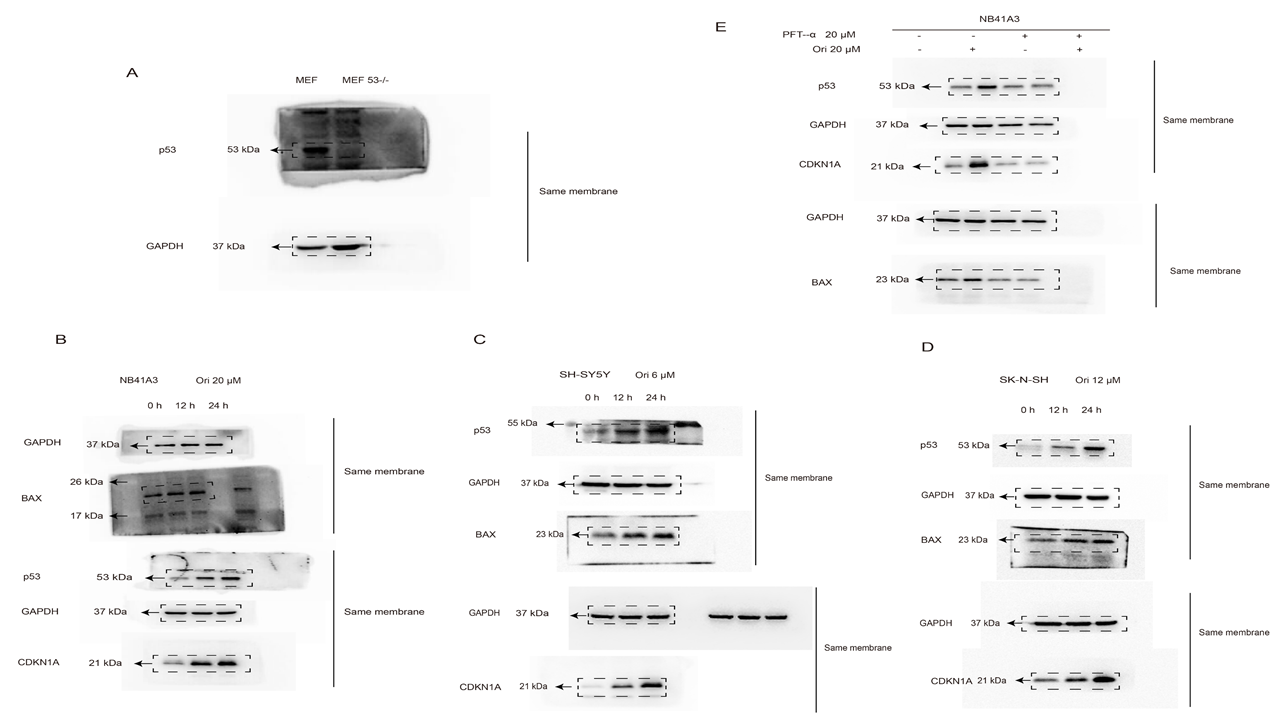

Supplement: Supplementary file 3 [file CAM4-8-5313-s003.tif]

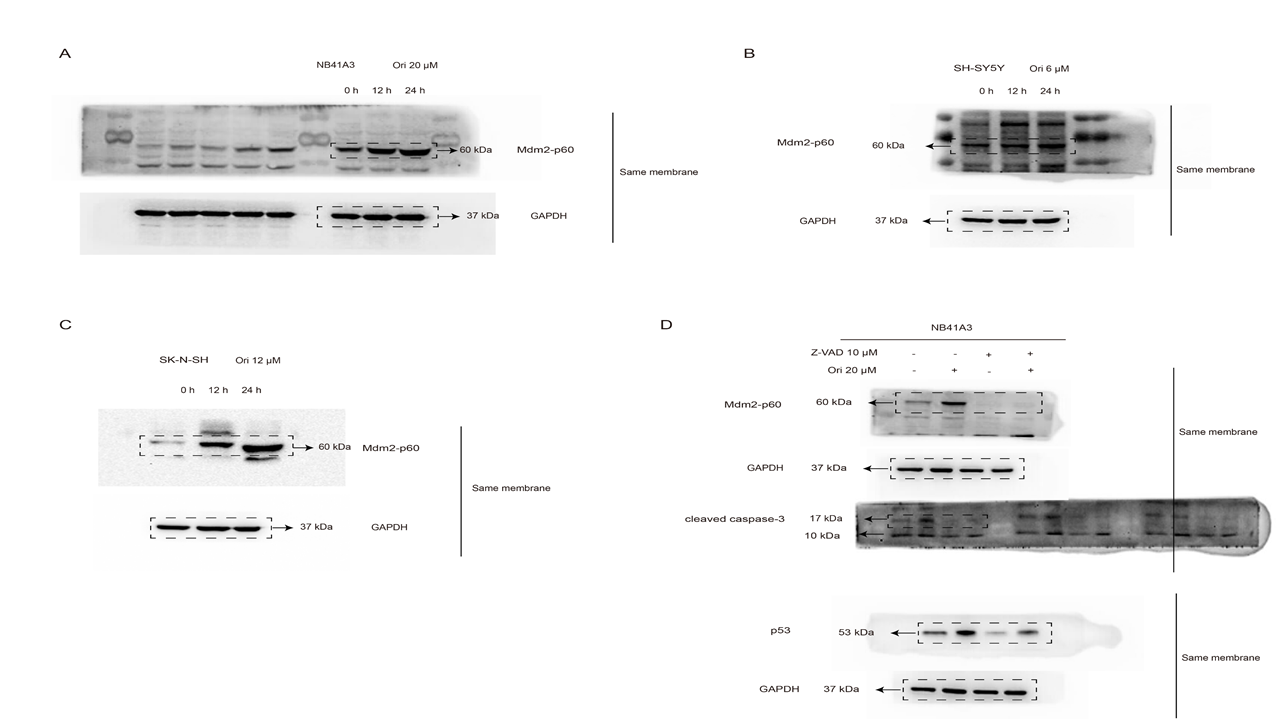

Supplement: Supplementary file 4 [file CAM4-8-5313-s004.tif]

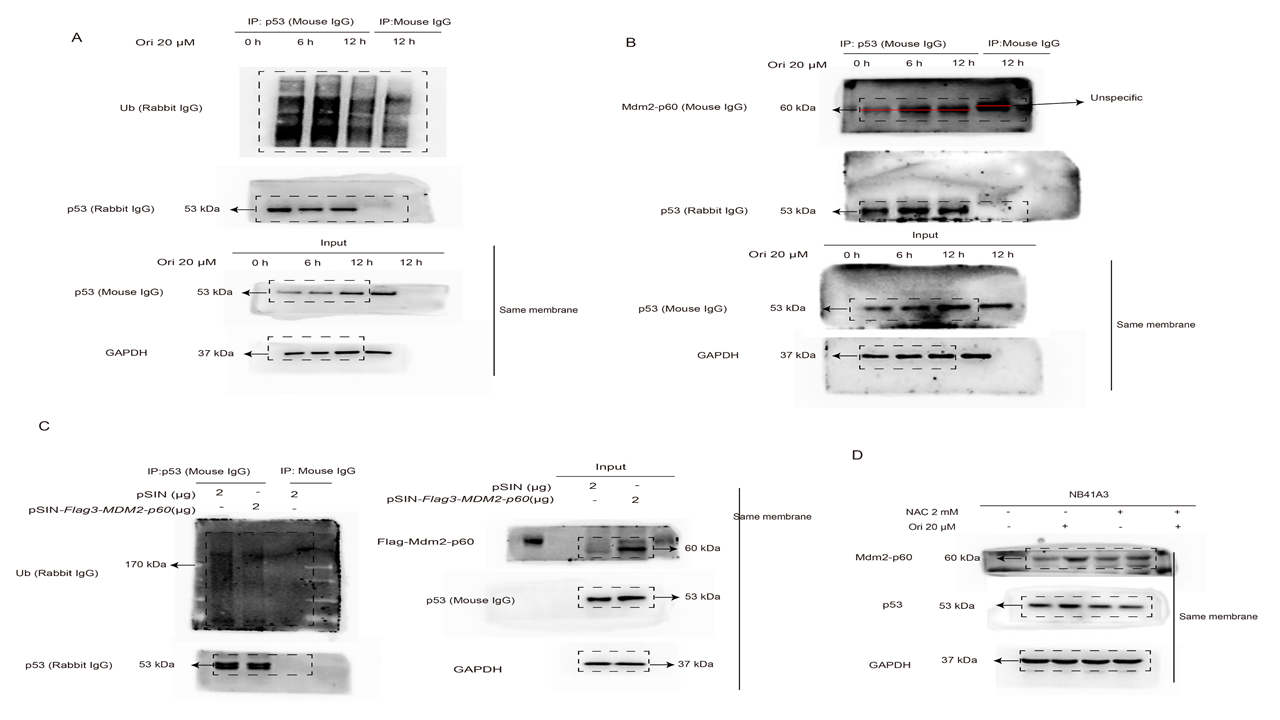

Supplement: Supplementary file 5 [file CAM4-8-5313-s005.tif]

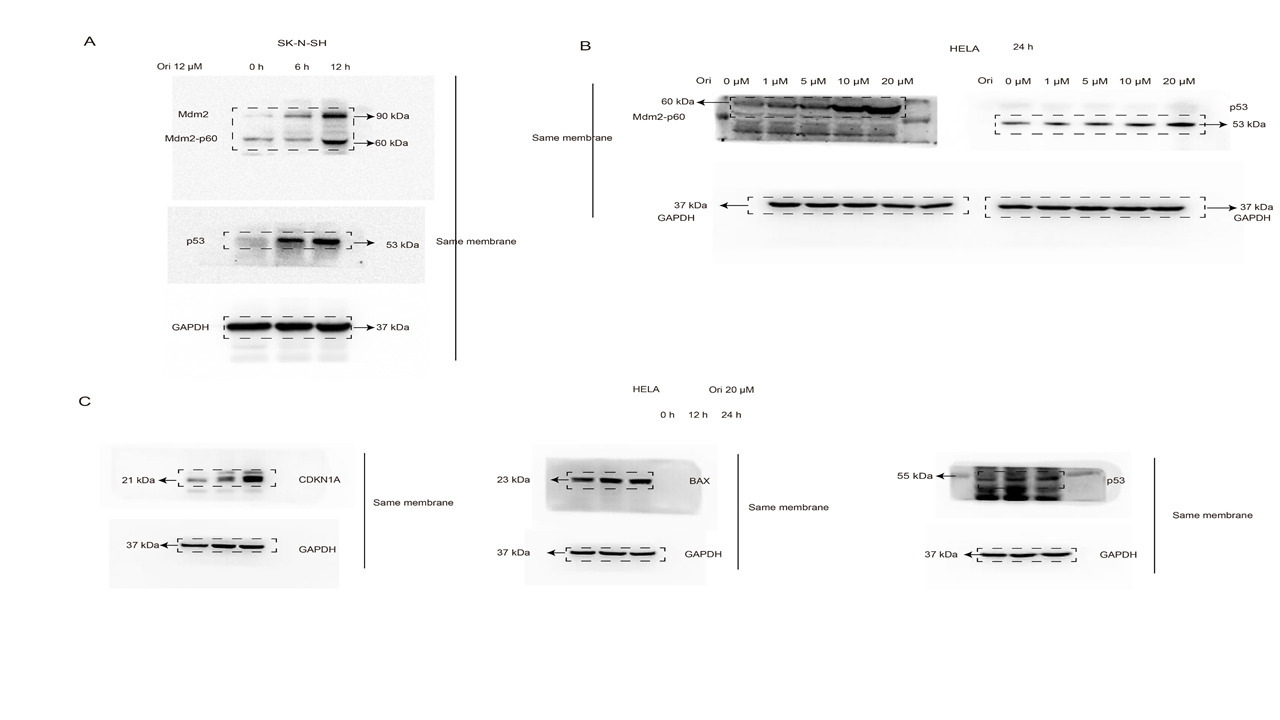

Supplement: Supplementary file 6 [file CAM4-8-5313-s006.tif]

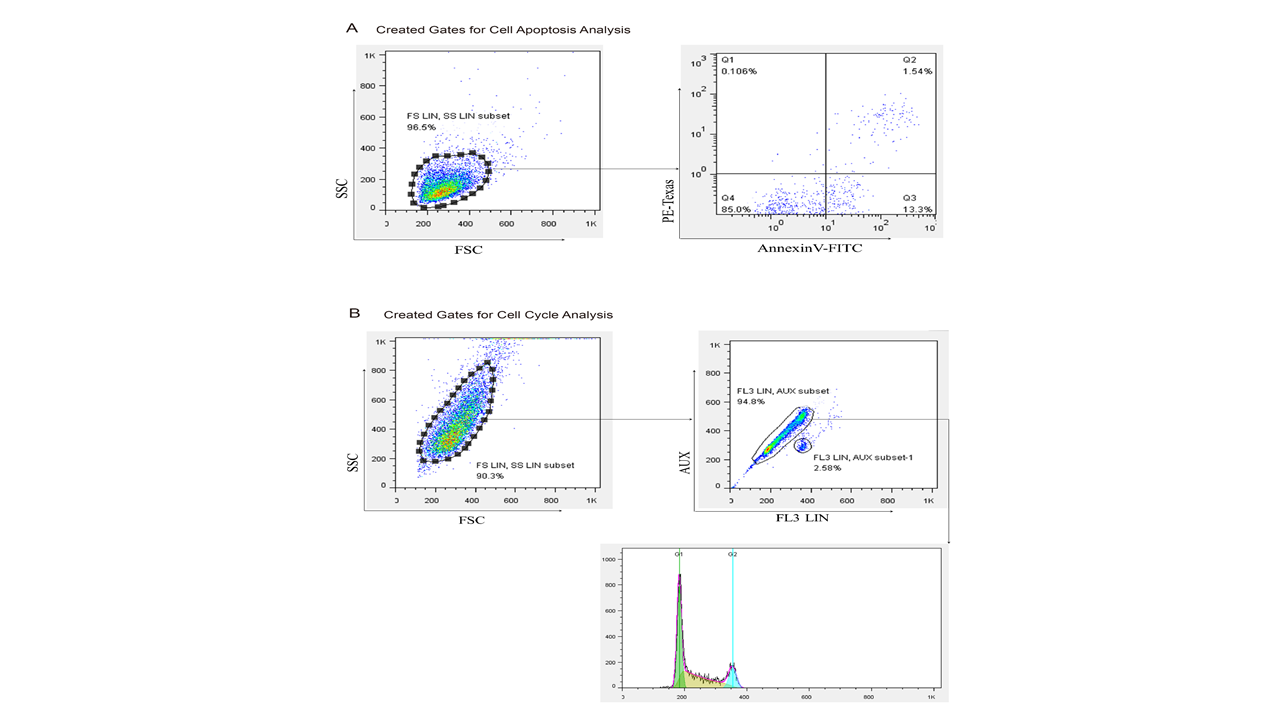

Supplement: Supplementary file 7 [file CAM4-8-5313-s007.tif]
